# Sycosis Menti

**Published:** 1892-05

**Authors:** 

**Affiliations:** Kiel, Germany


### SYCOSIS MENTI.

### Three Cases by Dr. Kunkel, of Kiel, Germany.

(Translated from *Archiv. fur Homœopathie,* by A. McNeil, M. D., San Francisco, California.)

Herr H., æt. thirty, consulted me April 22d, 1891. His mother had suffered much from nervous headache, but had been successfully treated by me with Sepia. He has suffered for several years from sycosis menti, which disfigured his pleasant face, and tormented him with intolerable itching which was better in the warmth of the bed. On his wrist an eruption had recently appeared (I did not describe it clearly in my journal). Since then his face is somewhat less affected. These were the concomitants of Sepia: "*First sweat, then aggravation of the itching and further aggravation in a warm room, better in the open air. Dullness and heaviness of the head on awaking in the morning, better by exercise. Aggravation of complaints by eating fat food, etc.*" These are well-known symptoms of that drug. I gave him Sepia$^{30}$, ten powders, one to be taken each week.

May 13th he reported "The eruption is clearly lessened. The itching had disappeared in three days. The breaking out on the wrist is dried up, that on the face is worse the last couple of days." Sepia$^{30}$, a powder every ninth evening.

July 22d. Improvement continues; small boils break out here and there which are becoming smaller and at longer intervals. A powder every two weeks.

October 28th. He reported that he was free from all itching and also from all the boils. I sent him Sepia$^{40}$ with directions to take a powder every ninth evening if a relapse should occur.

The formation of boils is a frequent accompaniment of artificial cures—*i.e.*, of homœopathic ones—and do not arise from any one remedy. Why should I expect a relapse? The formation of the pustules of barber's itch, it is well known, depends on the presence of a fungus. In many cases the eruption may be removed by remedies externally applied, such as a weak murcurial ointment. The constitutional disease on which it is based is of course untouched. The danger of relapses in these cases is much more imminent than when cured by internal remedies. However we will certainly succeed in averting a return if our patient, satisfied with the result attained, does not neglect the treatment prescribed. Such relapses teach us that those physicians are right who in the treatment of both acute and chronic diseases lay the chief weight on the treatment of the constitutional disease. Consequently we consider as irrational or at least insufficient the earlier attempts to discover agents capable of stopping the increase of fungi, and thereby destroying them if it could be done by innocuous means.

---

A teacher forty-six years old consulted me on the 22d of May, 1891. He had suffered much as a young child and also during his years at school from disease of the eyes. At that time he also suffered much from *headache when in school.* At the present *the air in the school-room is disagreeable to him* and *also sitting long at a time.* Sweet things aggravate, and *fat is repugnant.* For six weeks he has had sycosis menti. Periodical burning in the eruption; Sepia$^{30}$, eight powders, one every week.

June 30th. Substantial improvement; after the first powder the entire chin became covered with "a crust like white mold," there only remaining a trace in the mustaches. Sepia$^{30}$ at longer intervals. All trace of the disease soon gone.

---

G., a laborer of twenty-six years, consulted me in April, 1891. He says the members of his family suffer much from rheumatism. He has been tolerably healthy. For a couple of years he has suffered from barber's itch. His eyebrows are also afflicted, itching and burning, particularly during the new moon. During a severe east wind (in Germany a cold one) the skin of the face is dry (spröde). He always sleeps on the left side; muscles weak. Caust.$^{30}$, six powders, one every week.

May 15th. Decided improvement; the bad effects of the east wind less perceptible. Same prescription. [Why continue? McN.]

July 3d. On the 2d the symptoms increased. Same prescription.

August 28th. Health good, only at times he feels quite weak. Still sleeps on the left side. Caust.$^{200}$, a dose every week.

October 9th. His strength is now always good. Last week and a short time before violent itching spread over the whole body, particularly after becoming warm in bed, but that is gone; no further complaints.

Nov. 3d. I saw him again. Crises on the skin are, it is well known, desirable and not uncommon in the treatment of chronic diseases.

---

A sequala of scarlatina by Dr. Hesse, of Hamburg, Germany.

A child six years old had under my treatment scarlatina with diphtheria, which was followed by bright's disease with moderate dropsy. The renal disease had announced itself by vomiting, and after the albumen had disappeared from the urine the vomiting had continued and led to the following condition, which remained unchanged for almost fourteen days and seemed as if destined to cause the child's death. Sopor day and night only occasionally interrupted by the taking of nourishment, and more frequently by vomiting. Everything eaten comes up, and vomiting of mucus six or eight times a day, with frightful retching. Before vomiting great anxiety, restlessless, and symptoms which he could not describe further. After vomiting decided relief, no stool, urine dark, becoming cloudy and depositing a red sediment and smelling ammoniacal. Pulse remarkably slow, about fifty per minute.

The case puzzled me very much. The medicines I selected did not change the condition at all, and it was becoming every day more critical on account of the increasing weakness. On re-studying the case I took the relief after vomiting, and searched among the remedies which Bœnninghausen mentions with that symptom. Among these seven (Acon., Colch., Dig., Hyos., Nux-v., Puls., Sec-cor.) (to which I added Ipec. and Glon.) Digitalis attracted my attention on account of the slow pulse, which is so characteristic of that drug, and on consulting Hering and Jahr found it had the picture of my case: "Sopor interrupted by attacks of convulsive vomiting; vomiting day and night; persistent vomiting as if he would die; vomiting with relief of the complaints; pulse extremely slow; urine dark, becoming cloudy when standing: brick-dust sediment in the urine; ammoniacal smelling urine" (Bœnninghausen).

The child received five powders of Digitalis[30] to be taken morning and evening. Immediately after the first powder a radical change took place. The next day the sopor, vomiting (which occurred only once in the third day), and loss of appetite disappeared. He wanted to eat all the time, and the food was well borne. After the fourth powder the pulse was 80. Urine more abundant, pale yellow, no odor, no sediment, no further medicine was necessary.

Without doubt Digitalis was the simillimum, and in this case no other remedy could have done its work. This is the second time that I have administered Digitalis in my homœopathic practice. The first time also successfully (at least for several years). I reported years ago in the *Allg. Hom. Zeitung* a case of abdominal dropsy in which the remarkably slow pulse and the whitish stools guided me to the remedy.

The above case demonstrates once more that the general symptoms as sopor and vomiting cannot be taken as the leading ones for the choice of the remedy. Bœnninghausen gives Digitalis the fourth place (lowest) in sopor, and yet I found that it had that symptom so extremely important for my case, sopor interrupted by attacks of convulsive vomiting.

